# Mind–Body Exercises for Non-motor Symptoms of Patients With Parkinson’s Disease: A Systematic Review and Meta-Analysis

**DOI:** 10.3389/fnagi.2021.770920

**Published:** 2021-12-03

**Authors:** Kai Wang, Kunbin Li, Peiming Zhang, Shuqi Ge, Xiaopeng Wen, Zhiyuan Wu, Xianli Yao, Bing Jiao, Pingge Sun, Peipei Lv, Liming Lu

**Affiliations:** ^1^Department of Neurological Rehabilitation, Zhengzhou Central Hospital Affiliated to Zhengzhou University, Zhengzhou, China; ^2^Clinical Research and Data Center, South China Research Center for Acupuncture and Moxibustion, Medical College of Acu-Moxi and Rehabilitation, Guangzhou University of Chinese Medicine, Guangzhou, China; ^3^Department of Rehabilitation, Zhuhai Hospital of Integrated Traditional Chinese and Western Medicine, Zhuhai, China; ^4^Department of Medical Imaging, Zhengzhou Central Hospital Affiliated to Zhengzhou University, Zhengzhou, China

**Keywords:** mind–body exercises, global cognitive function, non-motor symptoms, meta-analysis, Parkinson’s disease

## Abstract

**Objective:** This study aimed to systematically evaluate the effects of mind–body exercise on global cognitive function, depression, sleep disorders, fatigue level, and quality of life (QOL) in a Parkinson’s disease (PD) population.

**Methods:** Total six English and Chinese databases were searched for articles published up to May 2021. Randomized controlled trials (RCTs) evaluating mind–body excises on non-motor symptoms of PD were included. The Cochrane risk of bias tool was used to assess the methodological quality, and we defined high-quality studies as having a low risk of bias in four or more domains. Global cognitive function was considered the primary outcome and was assessed using the Montreal Cognitive Assessment (MoCA). The secondary outcomes included QOL, fatigue, depression, and sleep quality, which were measured using the Parkinson’s Disease Questionnaire (PDQ-39), 16-item Parkinson’s Disease Fatigue Scale (PFS-16), Beck Depression Inventory (BDI), and revised Parkinson’s Disease Sleep Scale (PDSS-2), respectively. Subgroup analyses were conducted for global cognitive function and QOL to assess the optimal treatment measure across the various mind–body exercises.

**Results:** Fourteen RCTs with 404 patients were finally included in the meta-analysis. Eight (57.14%) studies were of high quality. The pooled results showed that mind–body exercises generally had a significant advantage over the control intervention in improving global cognitive function (MD = 1.68; *P* = 0.0008). The dose subgroup analysis revealed that the low dose (60–120 min per week) and moderate dose (120–200 min per week) significantly increased MoCA scores compared with the control group (MD = 2.11, *P* = 0.01; MD = 1.27, *P* = 0.02, respectively). The duration subgroup analysis indicated a significant difference in the effect of the duration (6–10 and >15 weeks) on increasing MoCA scores compared with the control group (MD = 3.74, *P* < 0.00001; MD = 1.45, *P* = 0.01, respectively).

**Conclusion:** Mind–body exercise may improve global cognitive function, sleep quality, and QOL in the PD population. In addition, low to moderate doses and appropriate durations significantly improved global cognitive function.

**Clinical Trial Registration:** [www.ClinicalTrials.gov], identifier [CRD42021275522].

## Introduction

Parkinson’s disease (PD) is a neurodegenerative movement disorder characterized by a range of motor symptoms, including resting tremor, rigidity, bradykinesia, and postural instability (Jankovic, 2008; Bega et al., 2014). However, various non-motor symptoms (NMSs), such as cognitive impairment, sleep disorders, fatigue, depression, anxiety, apathy, olfactory deficit, and constipation, can present at any stage of the disease, including early and premotor phases, finally leading to disability (Chaudhuri et al., 2006; Aarsland et al., 2010), and thus they are receiving increasing attention (Pfeiffer, 2016). Cognitive and psychiatric symptoms are the major non-motor features of PD (Meireles and Massano, 2012). Within 5 years of diagnosis, 25–50% of patients with PD develop to mild cognitive impairment (MCI) or dementia (Hershey and Peavy, 2015).

At present, the conventional treatment for PD is pharmacotherapy based on dopamine replacement. As the disease progresses, an increasing dosage of pharmacotherapy and drugs are usually ineffective, but simultaneously may cause side effects and limit physical and daily activities (Goldman and Weintraub, 2015). Moreover, many NMSs of PD are unresponsive to conventional pharmacotherapy. Effective alternative treatments for PD must be identified.

The National Center for Complementary and Alternative Medicine states that mind–body medicine is based on the interplay among the brain, mind, body, and behavior, as well as the powerful ways in which emotional, psychological, social, spiritual, and behavioral factors directly affect human health (Morone and Greco, 2007). Mind–body medicine focuses on respecting and enhancing each person’s self-knowledge and self-care abilities as a basic approach and highlights the techniques that underlie this approach (Health Information, 2006; Morone and Greco, 2007). Mind–body exercise as a non-pharmacological therapy has received increasing attention (Li et al., 2012; Amano et al., 2013; Gao et al., 2014; McGill et al., 2018; Cherup et al., 2021).

Previous meta-analyses of mind–body exercise have generally focused on the effects on the motor symptoms of patients with PD. Evidence for an improvement in NMSs of patients with PD is still insufficient. Among the various mind–body exercises, the effects on aspects of NMS are quite different (Ni et al., 2014; Zhang et al., 2019). Therefore, in this meta-analysis based on the current evidence, we combined five modalities (Tai Chi, yoga, Qigong, dance, and mindfulness meditation) of mind–body exercises to assess the effect on global cognitive function, sleep disorders, depression, fatigue, and quality of life (QOL) in the PD population.

## Methods

### Data Sources and Search Strategy

This meta-analysis adopted the Preferred Reporting Items for Systematic Reviews and Meta-Analyses (PRISMA) guidelines (Moher, 2009). Data were collected from PubMed, Embase, the Cochrane Library, the China National Knowledge Infrastructure (CNKI), the China Biology Medicine disc (CBM), and the Wanfang Database. These databases were searched systematically from inception to May 2021. The reference lists of the included studies were also manually searched to identify relevant articles. We further evaluated the obtained studies by reading the title and abstract to exclude studies that did not meet the criteria and then read the full text to determine eligibility. The detailed search strategy is described in Appendix 1.

### Study Selection

#### Types of Studies

All available randomized controlled trials (RCTs) evaluating mind–body excises (e.g., Tai Chi, Qigong, yoga, dance, and meditation) in the treatment of PD were considered, regardless of blinding, publication status, and trial duration. Non-randomized or quasi-randomized uncontrolled trials were excluded. Conference papers, animal experiments, case reports, conference papers, and some types of crossover study designs were not included.

#### Types of Participants

The target population with a diagnosis of idiopathic PD was defined using standard diagnostic criteria, such as the Movement Disorder Society (MDS) Clinical Diagnostic Criteria for PD (Postuma et al., 2015), the UK Parkinson’s Disease Society Brain Bank clinical diagnostic criteria (Rajput, 1993), diagnostic criteria for PD (Gelb et al., 1999), and the diagnostic criteria for idiopathic PD (Movement Disorders and Parkinson’s Disease Group, 2006). The population was included in the analysis regardless of age, sex, disease duration, Hoehn and Yahr (H&Y) stage, or disease severity.

#### Types of Interventions

Participants in the experiment group had to receive mind–body excise as the main intervention, including Tai Chi, yoga, Qigong, dance, meditation, or other forms of mind–body excises. The control groups included those who received usual care, no intervention, placebo, or routine physiotherapy exercises.

#### Types of Outcomes Measures

The primary outcome of interest was global cognitive function measured using the Montreal Cognitive Assessment (MoCA) (Nasreddine et al., 2005), which helps users identify cognitive deficits in domains including executive function, visual spatial skills, delayed recall memory, attention, conceptual thinking, calculation, language, and orientation (Litvan et al., 2011). Compared with the Mini-Mental State Examination (MMSE), the MoCA is a better measure of cognitive status in patients with PD without dementia, as it lacks both ceiling and floor effects (Biundo et al., 2016). The total MoCA score ranges from 0 to 30 points, and a score >26 is considered within the normal range. Higher scores indicate better cognitive performance.

The secondary outcomes included the Parkinson’s Disease Questionnaire (PDQ-39), 16-item Parkinson’s Disease Fatigue Scale (PFS-16), Beck Depression Inventory (BDI), and revised Parkinson’s Disease Sleep Scale (PDSS-2) scores. QOL was measured using the PDQ-39, which comprises 39 questions covering 8 dimensions (including mobility, activities of daily living, mental well-being, stigma, social support, cognition, communication, and physical discomfort). It is a widely used patient health questionnaire specifically for PD (Rodrigues et al., 2007). Lower scores indicate better performance. The PFS-16 is used to evaluate fatigue and was specifically designed for patients with PD. The PFS-16 aims to measure the physical fatigue of patients with PD and its effect on patients’ daily living abilities. It does not examine cognitive and emotional problems related to fatigue, with good reliability, specificity (82.1%) and sensitivity (84.7%) (Friedman et al., 2010). The PFS-16 contains 16 items, each of which is scored from 1 to 5 points. The sum of all items divided by the total number of the 16 items is the average of the fatigue score (the cutoff point for the fatigue and non-fatigue groups is 3.3, and a mean PFS-16 score greater than 3.3 is defined as fatigue). Lower scores represent less fatigue (Brown et al., 2005). We used the BDI to assess the severity of depressive symptoms in patients with PD. The BDI is a questionnaire containing 21 items that measures symptoms within the previous 2 weeks (Beck et al., 1961). The higher the total score, the more severe the depressive symptoms. PDSS-2 is used to assess the sleep quality. This 15-item questionnaire surveys sleep problems concerned with motor symptoms at night, PD symptoms at night, and disturbed sleep. The total score ranges from 0 to 60 points. Lower scores indicate better sleep quality (Trenkwalder et al., 2010).

### Data Extraction

Two independent investigators (KW and SG) performed data extraction from the eligible studies using a collection form. The following basic information was extracted from the included studies: (1) the characteristics of each study: year of publication, author, study group, and country; (2) population: age, sex, and duration of disease sample size; (3) intervention and control groups: type, duration, and frequency; and (4) main outcomes: the means and standard deviations (means ± SD) of the primary and secondary outcomes. If the data in the studies were incomplete, we contacted the corresponding authors by e-mail. Any discrepancies were resolved by discussion.

### Quality Assessment

Two independent investigators (KW and SG) assessed the methodological quality of the studies using the Cochrane risk of bias assessment (version 5.1.0) (Higgins and Altman, 2011), which covers seven domains: random sequence generation; allocation concealment; blinding of patients, therapist, and outcome assessors; incomplete outcome data; selective reporting; and other sources of bias. Each bias domain was judged as low, unclear, or high. Studies were defined as high quality if they had a low risk of bias in four or more domains. Discrepancies between the reviewers were resolved through discussion.

### Data Analysis

We performed our meta-analysis using Review Manager software 5.3 (Cochrane Collaboration, Oxford, United Kingdom). For all the outcome measures we used that were continuous variables, the mean difference (MD) or standardized mean difference (SMD) with a 95% confidence interval (CI) was calculated. The SMD statistic was selected to evaluate the results of different scales. *I*^2^ statistics were used to assess the heterogeneity and to choose the effect model. If *I*^2^ ≤ 50% and *P* ≥ 0.1, the included studies were considered homogeneous, and a fixed-effects model was selected. Otherwise, if *I*^2^ > 50% and *P* < 0.1, indicating that statistical heterogeneity existed among studies, a random-effects model was selected. In this meta-analysis, *P* < 0.05 was considered statistically significant.

If clinical heterogeneity was present in the combined results, a subgroup analysis was performed to identify the source of heterogeneity. Meanwhile, we conducted subgroup analyses to explore the effect of different categories of mind–body exercise. For the primary outcome (the global cognitive function), subgroup analyses were performed for the different intervention categories (Tai Chi, yoga, Qigong, dance, and meditation), the frequency (low dose, moderate dose, and high dose), and the duration (5, 6–10, 11–15, and >15 weeks).

We also performed a sensitivity analysis by sequentially eliminating each study to test the stability of the results. Before calculating the effect size, we deleted each of the included studies and excluded those that resulted in high heterogeneity or altered the pooled effect of the results. For the primary outcomes, we used Egger’s test to evaluate the publication bias of the included studies.

## Outcomes

### Study Identification and Selection

**FIGURE 1:**
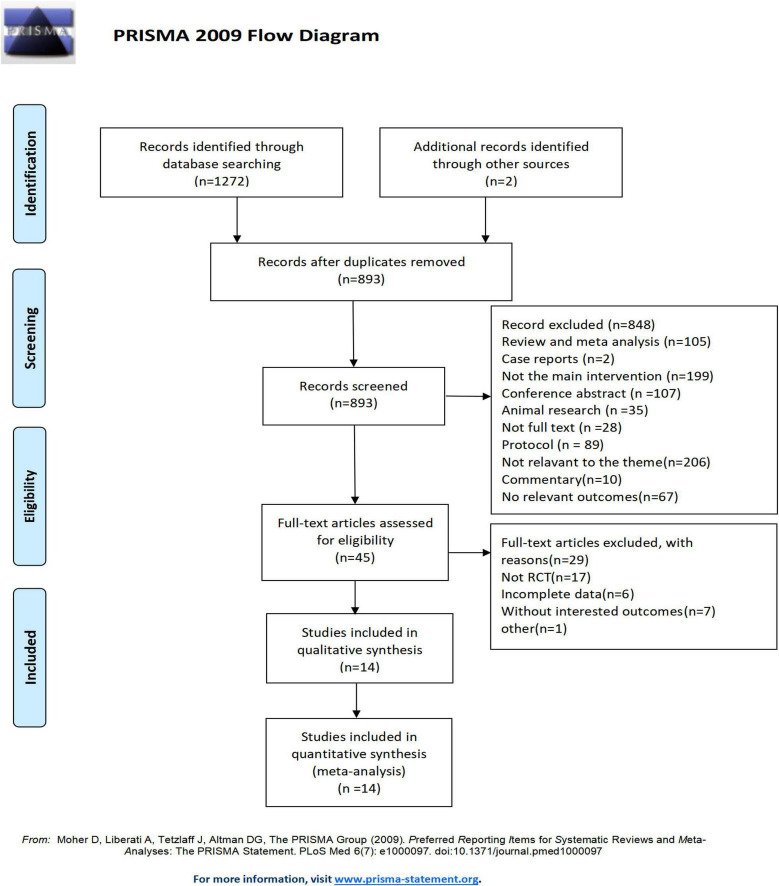
Flow of the trial selection process.

A total of 1272 relevant records were initially identified through database searches, and 2 additional records were identified through other sources. After removing duplicate entries, 381 records were excluded. After screening the titles and abstracts of the studies, 848 were excluded. After full-text screening, only 14 RCTs (Nocera et al., 2013; Volpe et al., 2013; Romenets et al., 2015; Ni et al., 2016; Fan et al., 2017; Moon et al., 2017, 2020; Cheung et al., 2018; Michels et al., 2018; Son and Choi, 2018; Wu et al., 2018; Solla et al., 2019; Zhu et al., 2020; Frisaldi et al., 2021) met the inclusion criteria and were finally included in the quantitative synthesis. The detailed process of identification and selection is shown in the PRISMA flow diagram (Figure 1).

### Study Characteristics

The characteristics of the included studies are summarized in Table 1. Four hundred four participants diagnosed with PD of mild to moderate severity were included. The sample size ranged from 10 to 66. The articles were published in Chinese or English. Eleven (78.5%) included studies were published between 2016 and 2021. The countries of the publications were the United States (*n* = 6, 42.85%), China (*n* = 3, 21.42%), Italy (*n* = 3, 21.42%), Canada (*n* = 1, 7.14%), and Korea (*n* = 1, 7.14%).

**TABLE 1:** Characteristics of randomized controlled trials included in the meta-analysis.

References, country	Mean age, year	Duration of disease, year	Hoehn–Yahr	No. T/C	Sex, M/F	Experimental group Intervention	Control group Intervention	Duration	Outcome assessments
Zhu et al., 2020, China	T: 68.53 ± 1.90 C: 67.77 ± 1.72	T: 4.68 ±0.43 C: 4.00 ± 0.39	T: 2 (2.2) C: 2 (1.2)	19/22	T: 12/7 C: 13/9	Tai Chi training + routine exercise Frequency: 40–50 min, five times a week (200–250 min per week)	Routine exercise	12 weeks	PDQ-39, MoCA
Michels et al., 2018, United States	T: 66.44 C: 75.50	N	T: 2.11 (0.33) C: 2.50 (1.00)	9/4	N	Dance therapy Frequency: 60 min/week	No treatment	10 weeks	MoCA, BDI, PDQ-39
Moon et al., 2020, United States	T: 66.4 (8.1) C: 65.9 (5.4)	T: 4.25 (2.1) C: 5.33 (3.3)	T: 2 (2–2) C: 2 (2–2)	8/9	T: 4/4 C: 3/6	Qigong group Frequency: 15–20 min twice a day +45 min^–^1 h of group exercise/week (255–340 min per week)	Sham Qigong	12 weeks	PDQ-39, PFS-16, PDSS-2
Cheung et al., 2018, United States	T: 63.5 (8.5) C: 65.8 (6.6)	4.8 (2.9)	T: 2 (0.8) C: 2 (0.8)	10/10	N	Hatha yoga program Frequency: 60 min per session twice weekly (120 min per week)	No treatment	12 weeks	MoCA, BDI
Nocera et al., 2013, United States	T: 66 (11) C: 65 (7)	T: 8.08 (5.4) C: 6.83 (1.83)	2–3	15/6	T: 7/8 C: 4/2	Tai Chi sessions Frequency: 60 min, three times per week (180 min per week)	No treatment	16 weeks	PDQ-39
Romenets et al., 2015, Canada	T: 63.2 (9.9) C: 64.3 (8.1)	T: 5.5 (4.4) C: 7.7 (4.6)	1–3	18/15	T: 12/6 C: 7/8	Argentine tango Frequency: 1 h, twice a week (120 min per week)	Self-directed exercise	12 weeks	PDQ-39, MoCA, BDI
Solla et al., 2019, Italy	T: 67.8 ± 5.9 C: 67.1 ± 6.3	T: 4.4 ± 4.5 C: 5 ± 2.9	T: 2.1 ± 0.6 C: 2.3 ± 0.4	10/9	T: 6/4 C: unclear	Sardinian folk dance program Frequency: 90 min/session, two sessions/week (180 min per week)	Usual care	12 weeks	PFS-16, BDI-II, MoCA
Volpe et al., 2013, Italy	T: 61.6 ± 4.5 C: 65.0 ± 5.3	T: 9.0 ± 3.6 C: 8.9 ± 2.5	T: 2.2 ± 0.4 C: 2.2 ± 0.4	12/12	T: 7/5 C: 6/6	Irish set dancing Frequency: 90 min, once per week (90 min per week)	Standard physiotherapy exercises	6 months	PDQ-39
Ni et al., 2016, United States	T: 71.2 (6.5) C: 74.9 (8.3)	T: 6.9 (6.3) C: 5.9 (6.2)	T: 2.2 ± 0.7 C: 2.1 ± 0.7	13/10	N	Yoga program Frequency: 60 min/session, two sessions/week (120 min per week)	Usual care	12 weeks	PDQ-39
Frisaldi et al., 2021, Italy	T: 60.68 (6.34) C: 61.2 (7.18)	T: 5.99 (2.18) C: 6.43 (2.50)	T: 2 C: 2	19/19	T: 13/6 C: 10/9	Dance-physiotherapy combined intervention Frequency: 1 h conventional physiotherapy +1 h of dance class, three times a week (360 min per week)	2 h of conventional physiotherapy	5 weeks	MoCA, BDI, PFS-16
Moon et al., 2017, United States	T: 61.8 ± 5.7 C: 68.0 ± 5.3	T: 8.0 ± 3.6 C: 13.3 ± 3.6	T: 2.7 ± 0.3 C: 2.4 ± 0.5	5/5	N	Qigong Frequency: 15–20 min/session, twice per day (210–280 min per week)	Sham Qigong	6 weeks	PDSS-2
Son and Choi, 2018, Korea	<60: T: 6.1% C: 10% 60–60: T: 51.5% C: 70% ≥70: T: 42% C: 20%	N	N	33/33	N	The mindfulness meditation-based complex exercise program Frequency: 2 h, once a week (120 min per week)	Usual care	8 weeks	MoCA, PDSS-2
Fan et al., 2017, China	64.06 ± 8.56	N	1–3	18/18	N	Qigong Frequency: 60 min, five times per week (300 min per week)	Usual care	8 weeks	MoCA
Wu et al., 2018, China	T: 62.42 ± 5.37 C: 64.66 ± 5.47	T: 4.75 ± 2.01 C: 4.25 ± 1.96	1–3	28/24	T: 20/8 C: 17/7	Tai Chi Frequency: 40 min, four times a week (160 min per week)	Routine exercise	16 weeks	MoCA, PDQ-39

The intervention investigated in the trials included Tai Chi (*n* = 3, 21.42%), dance (*n* = 5, 35.71%), yoga (*n* = 2, 14.29%), Qigong (*n* = 3, 21.42%), and the mindfulness meditation-based complex exercise program (MMBCEP, *n* = 1, 7.14%). The intervention duration ranged from 5 to 16 weeks, with a frequency ranging from 60 to 300 min per week. Four (28.57%) of the control groups were treated with routine excise, 4 (28.57%) with usual care, 3 (21.42%) with no intervention, 2 (14.29%) with placebo, and 1 (7.14%) with self-directed excise.

Nine (64.29%) trials selected the MoCA to assess global cognitive function. Eight (57.14%) trials used the PDQ-39 to assess QOL. Three (21.42%) trials used the PDSS-2 to assess sleep quality. Three (21.42%) trials used PFS-16 to assess the fatigue level. Five (35.71%) trials used the BDI to assess the severity of depression.

### Quality Assessment

**FIGURE 2:**
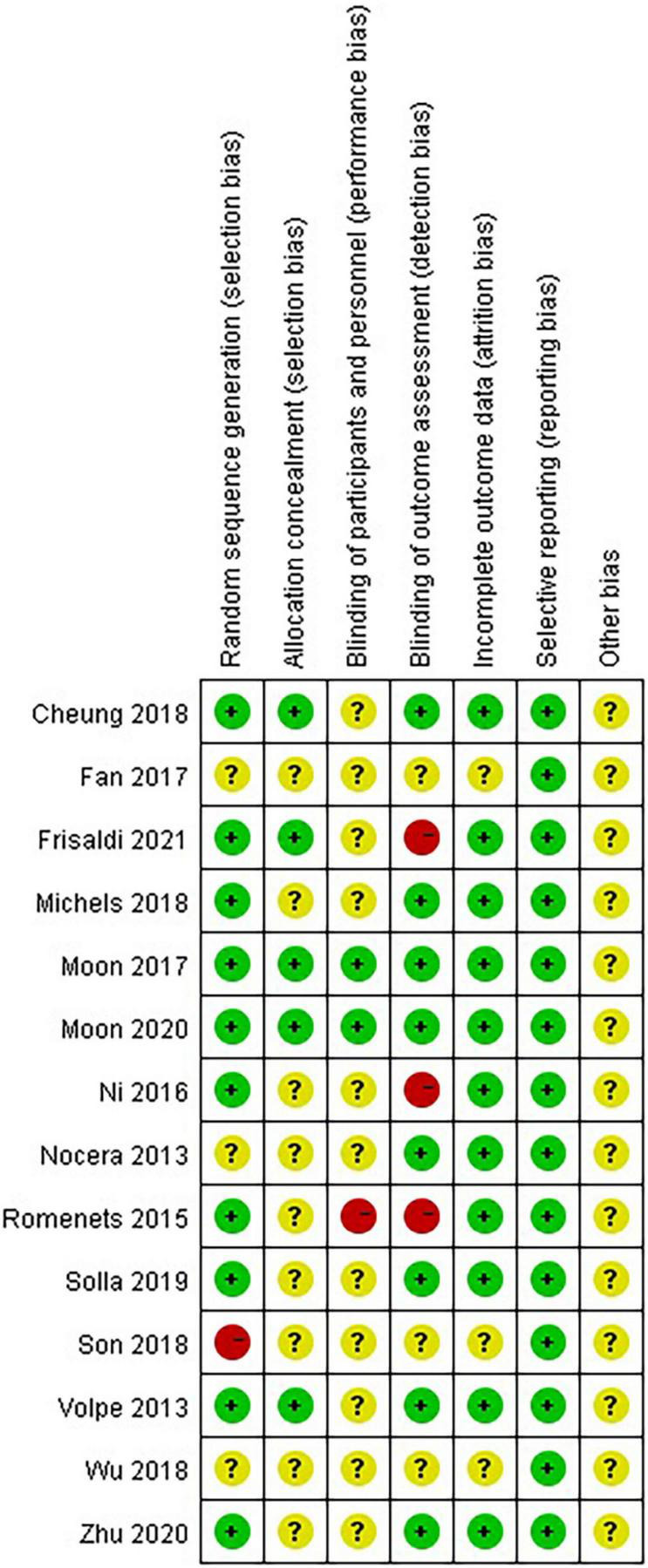
Risk of bias summary: review authors’ judgments about each risk of bias item for each included study.

**FIGURE 3:**
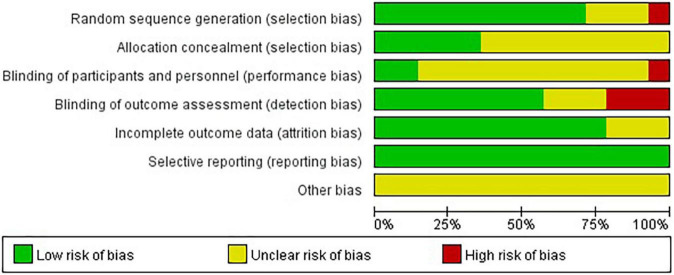
The risk of bias for the included studies.

Eleven studies (78.57%) reported the methods of random sequence generation. Seven studies (50.00%) used computer-generated random numbers, 3 (21.42%) used block or stratified randomization, and 1 (7.14%) used a random number table. Five studies (35.71%) reported the use of allocation concealment methods. Only two studies (14.29%) succeeded in blinding the participants and personnel. Eight studies (57.14%) were assessed as having a low risk of bias considering the blinding of assessors. Eleven studies (78.57%) reported a low risk of attrition bias. Regarding reporting bias, we judged that all the studies stated the expected results. Most of the studies did not describe other risks of bias. Eight (57.14%) studies were defined as high quality. A summary of the quality assessment of the fourteen RCTs is presented in Figures 2, 3.

### Effects of the Intervention

#### Primary Outcomes

##### Global Cognitive Function (Montreal Cognitive Assessment)

###### Meta-Analysis

**FIGURE 4:**
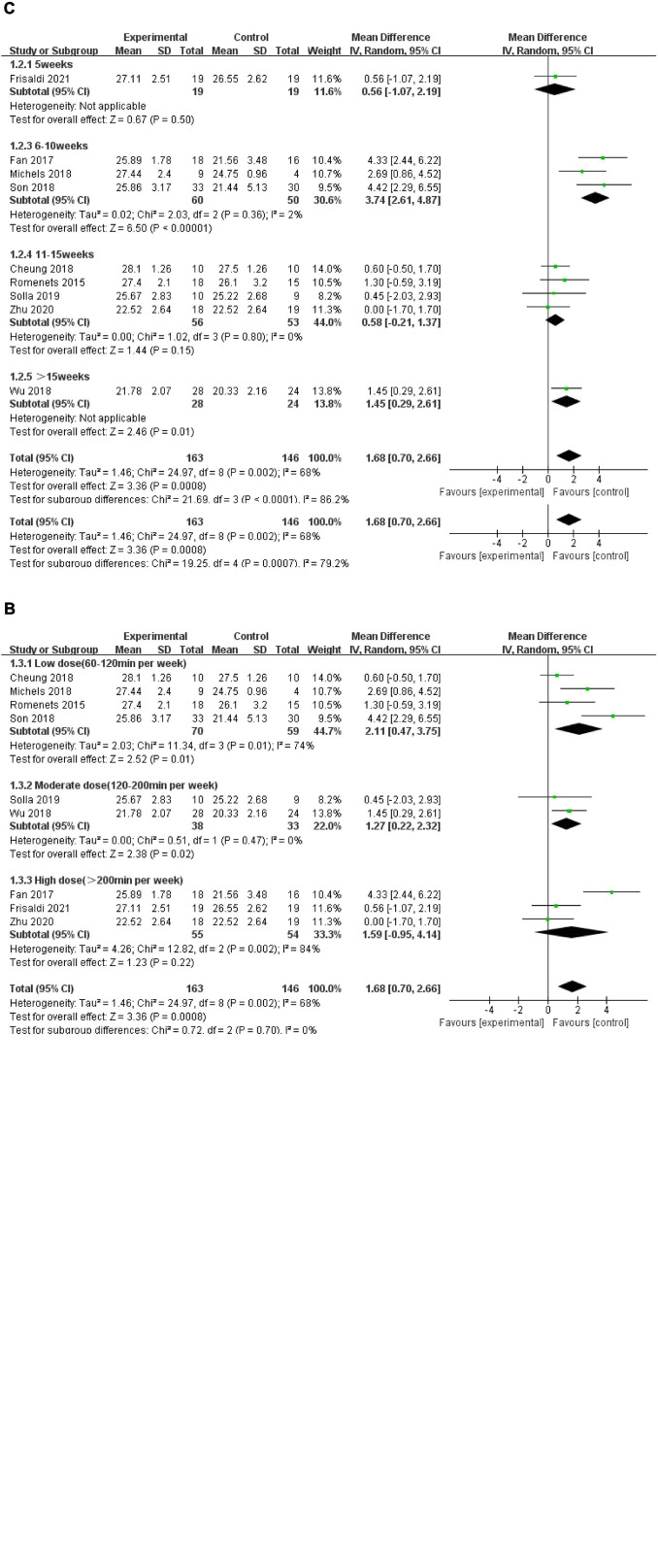
**(A)** Forest plot showing the effects of mind–body exercises on global cognitive function. **(B)** Forest plot showing the effects of mind–body exercises on global cognitive function in subgroups stratified according to the dose of the intervention. **(C)** Forest plot showing the effects of mind–body exercises on global cognitive function in subgroups stratified according to the different durations of the intervention.

Nine included RCTs with 309 patients using MoCA as the measurement were included in the meta-analysis to evaluate global cognitive function. A random-effects model was used, as high heterogeneity existed (*P* = 0.002, *I*^2^ = 68%). The combined results suggested that these five types of mind–body exercises had a significant advantage over the control group in improving global cognitive function (MD: 1.68, 95% CI: 0.70–2.66, *P* = 0.0008). The pooled data from four studies showed that dance significantly increased MoCA scores compared with the control group (MD: 1.30, 95% CI: 0.28–2.32, *P* = 0.01). Two studies examining Tai Chi did not show advantages in global cognitive function compared with the control group (MD: 0.87, 95% CI: −0.53 to 2.26, *P* = 0.22). One Qigong (MD: 4.33, 95% CI: 2.44–6.22, *P* < 0.00001), and one MMBCEP (MD: 4.42, 95% CI: 2.29–6.55, *P* < 0.0001) study each separately formed a subgroup, showing a significant difference in increasing MoCA scores. However, only one study examining yoga did not show a significant difference in improving global cognition (MD: 0.60, 95% CI: −0.50 to 1.70, *P* = 0.29) (Figure 4A).

###### Subgroup Analysis

Since the aggregated MoCA scores exhibited high heterogeneity (*P* = 0.002, *I*^2^ = 68%), subgroup analyses were performed based on the various characteristics of the intervention, such as the frequency, duration, and intervention provided to the control group, to determine the sources of heterogeneity.

Subgroup analysis based on different frequencies of the intervention: we determined the total number of minutes weekly that the mind–body excises were conducted in each study and classified them into a low dose (60–120 min per week), moderate dose (120–200 min per week), and high dose (>200 min per week). No significant differences were observed among the subgroups (*P* = 0.70, *I*^2^ = 0%). The subgroup analysis revealed that both the low-dose intervention (MD: 2.11, 95% CI: 0.47–3.75, *P* = 0.01) and moderate dose intervention (MD: 1.27, 95% CI: 0.22–2.32, *P* = 0.02) significantly increased MoCA scores compared with the control group. However, the high-dose intervention showed no improvement in MoCA scores (MD: 1.59, 95% CI: −0.95 to 4.14, *P* = 0.22) (Figure 4B).

Subgroup analysis based on different durations of the intervention: The intervention period of the nine studies ranged from 5 to 16 weeks. We classified the duration of the intervention into 5, 6–10, 11–15, and >15 weeks. Significant differences were observed among the subgroups (*P* < 0.0001, *I*^2^ = 86.2%). The duration (6–10 weeks) of mind–body excise in three articles resulting in a significant difference in increasing MoCA scores compared with control group (MD: 3.74, 95% CI: 2.61–4.87, *P* < 0.00001). Meanwhile, only one study with a duration longer than 15 weeks revealed a significant effect on increasing MoCA scores (MD: 1.45, 95% CI: 0.29–2.61, *P* = 0.01). However, only one study with an intervention period of 5 weeks showed no significant difference (MD: 0.56, 95% CI: −1.07 to 2.19, *P* = 0.50). The pooled results of four studies with intervention durations ranging from 11 to 15 weeks also showed no significant difference in improving MoCA scores compared with the control group (MD: 0.58, 95% CI: −0.21 to 1.37, *P* = 0.15) (Figure 4C).

###### Sensitivity Analysis

We performed a sensitivity analysis by sequentially eliminating each study and found that the combined results were stable and not affected by a single dataset (Supplementary Figure 1).

#### Secondary Outcomes

##### Sleep Quality (Parkinson’s Disease Sleep Scale)

**FIGURE 5:**
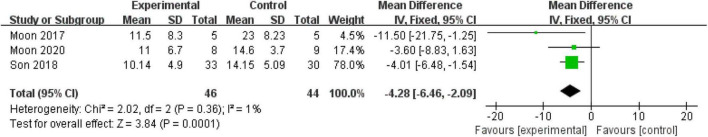
Forest plot showing the effects of mind–body exercises on sleep quality.

Three (21.43%) included RCTs with 90 patients measured sleep quality using the PDSS-2 scale. A fixed-effect model was used since no heterogeneity existed (*P* = 0.36, *I*^2^ = 1%). The pooled result showed that mind–body excise had a significant effect on improving the sleep quality of patients with PD (MD: −4.28, 95% CI: −6.46 to −2.09, *P* = 0.0001) (Figure 5).

##### Fatigue (Parkinson’s Disease Fatigue Scale)

**FIGURE 6:**
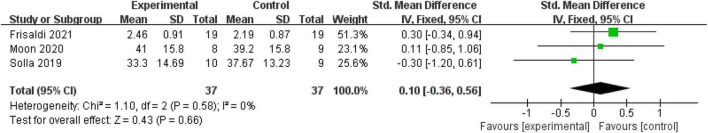
Forest plot showing the effects of mind–body exercises on fatigue.

Three (21.43%) included RCTs with 74 patients used PFS-16 to assess the degree of fatigue. Because two studies (Solla et al., 2019; Moon et al., 2020) used the total PFS-16 score, and one (Frisaldi et al., 2021) used the average score of 16 items. We calculated the SMD to eliminate the difference. A fixed-effect model was used since no heterogeneity existed (*P* = 0.58, *I*^2^ = 0%). However, the pooled result showed that mind–body excise had no significant effect on improving the fatigue level of patients with PD (SMD: 0.10, 95% CI: −0.36 to 0.56, *P* = 0.66) (Figure 6).

##### Depression (Beck Depression Inventory)

**FIGURE 7:**
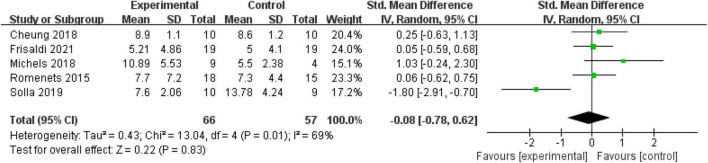
Forest plot showing the effects of mind–body exercises on depression.

Five (35.71%) RCTs with 123 patients assessed the depressed mood using the BDI. High heterogeneity existed in the included studies (*P* = 0.01, *I*^2^ = 69%); thus, a random-effect model was used. The meta-analysis showed that mind–body excise did not induce a significant difference in relieving the symptoms of depression in the PD population (MD: −0.08, 95% CI: −0.78 to 0.62, *P* = 0.83) (Figure 7).

##### Quality of Life (Parkinson’s Disease Questionnaire)

**FIGURE 8:**
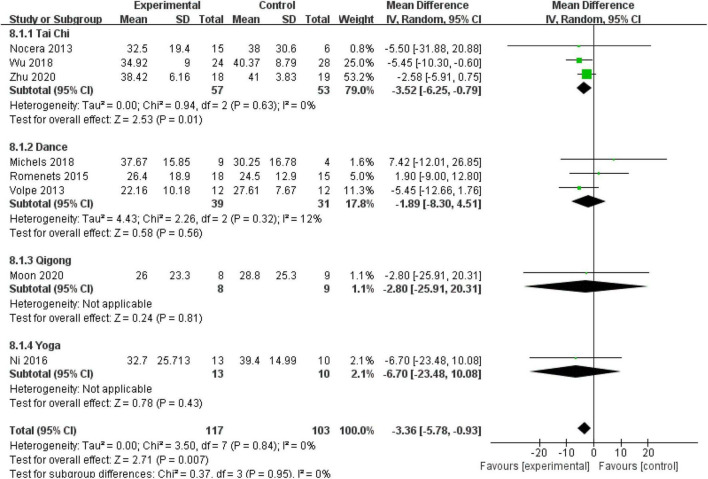
Forest plot showing the effects of mind–body exercises on quality of life.

Eight (57.14%) RCTs with 220 patients assessed the QOL using the PDQ-39. No heterogeneity was observed (*P* = 0.84, *I*^2^ = 0%), and a fixed-effect model was used. The aggregated result showed that the mind–body excise group generally presented improved PDQ-39 scores compared with the control group (MD: −3.36, 95% CI: −5.78 to −0.93, *P* = 0.007). No significant differences were observed among the subgroups (*P* = 0.95, *I*^2^ = 0%). The subgroup analysis indicated that the Tai Chi group was more sensitive to improving PDQ-39 scores than the other groups (MD: −3.52, 95% CI: −6.25 to −0.79, *P* = 0.01) (Figure 8).

### Publication Bias

Based on Egger’s test, no significant publication bias was observed in studies evaluating global cognitive function (Egger’s test *P* = 0.275) (Supplementary Table 2).

### Adverse Events and Safety

Nine studies (64.28%) discussed adverse events, five (31.25%) of which reported that no adverse events occurred. Four (28.57%) studies discussed the details of adverse events: two (14.28%) were related to mild fatigue and muscle cramps, one (7.14%) was non-injurious fall for which participants did not medical attention, and one (7.14%) was mild low back pain that was not clearly determined to be related to treatment. No serious adverse events related to mind–body exercises were reported. Overall, all types of mind–body exercises were well tolerated and relatively safe.

## Discussion

### Summary of Findings

We performed a meta-analysis of 14 RCTs with 404 participants. The meta-analysis indicated mind–body excises generally resulted in significant improvements in the outcomes: MoCA, PDSS-2, and PDQ-39 scores. For the primary outcome, the subgroup analysis showed that Qigong, dance, and MMBCEP might be more sensitive to improving global cognitive function. In addition, we concluded that a low to moderate dose (60–200 min per week) of mind–body exercise with a duration of 6–10 or >15 weeks potentially improved MoCA scores. Regarding the secondary outcomes, among the various mind–body excises, we recommended Tai Chi to improve PDQ-39 scores.

### Mechanisms of Mind–Body Exercise

Tai Chi and Qigong, two forms of traditional Chinese exercises, combine body movements, breathing and mental training to maintain health and eliminate disease syndromes. Previous reviews have provided evidence that Tai Chi is beneficial for specific diseases, such as PD, cognitive disorder, dementia, and depression, and focus on the potential mechanisms of Tai Chi in modulating brain morphology, functional homogeneity, activity, and connectivity (McCarville, 2016). Some studies observed changes in the cognitive potential P300 before and after Qigong therapy as an adjuvant to clinical treatment and found that the N2 and P3 latent periods of ERPs in patients with PD were significantly shortened after Qigong exercise, reflecting improvements in the cognitive processes of attention control and processing ability, which confirmed the efficacy of Qigong exercise as a supplementary treatment to PD (Yu et al., 1998; Song et al., 2003), Mindfulness MMBCEP is an integrative program that combines mindfulness stress reduction (MBSR) with the senior fitness test manual, which alleviate depression and anxiety, helping people eliminate negative thought and relax, and subsequently exerting beneficial effects on sleep (Jones and Rikli, 2002). Dance therapy is a form of enjoyment, motivation and participation in sports rehabilitation for patients through which people creatively engage in a process that further promotes their emotional, cognitive, physical, and social integration (Meekums et al., 2012). Dance therapy improves the psychological state of patients with PD and exerts a positive effect on brain neuroplasticity (Kattenstroth et al., 2010). Yoga practice, a popular mind–body exercise concentrating on breathing, meditation, and postures, has been reported to improve muscle strength, endurance, balance, flexibility, and healthy well-being (Lim and Cheong, 2015).

### Compared to Related Studies

To the best of our knowledge, this comprehensive systematic review and meta-analysis is the first to evaluate the effect of mind–body excise on the various NMSs of the PD population, including global cognitive function, sleep quality, fatigue, depression, and QOL. Previous meta-analyses mainly focused on the effects of one or two certain types of mind–body exercise on the motor function of patients with PD, with little or no reference to NMSs (Sharp and Hewitt, 2014; Yang et al., 2015; Delabary et al., 2018; Jin et al., 2019). In our review, we comprehensively analyzed the effects of a range of mind–body excises, such as Tai Chi, Qigong, dance, yoga, and mindfulness meditation, to provide some reference for clinical practice.

Our combined analysis of the effects of mind–body excises on MoCA scores indicated that compared with the control group, mind–body excises had obvious advantages in improving global cognitive function. High heterogeneity existed in the combined results. We conducted subgroup analyses to determine the source of heterogeneity. Based on the type of mind–body excises, the subgroup analysis indicated that Qigong, dance, and MMBCEP might be more sensitive to improving the global cognition function of patients with PD presenting with or without cognitive deficits, which was inconsistent with the previous meta-analysis by Zhang et al. (2019) found that dance did not effectively improve global cognitive function, except for executive function, but the review lacked a sufficient number of RCTs. In our analysis, Tai Chi and yoga did not significantly improve the MoCA scores, potentially due to the limited number of trials. The subgroup analysis stratified according to the frequency of intervention suggested that a low dose (60–120 min per week) and moderate dose (120–200 min per week) were effective at improving global cognition. We did not find any other meta-analysis that discussed the effect of different frequencies of mind–body excise on improving cognitive function in patients with PD. However, our result was consistent with a previous review showing that a moderate dose (60–120 min per week) might be the optimal mind–body exercise dose for improving cognitive function in older adults (Wu et al., 2019). A high-dose (>200 min per week) intervention did not exert an obvious effect on the global cognitive function of patients with PD, which might cause physical fatigue and prevent them from performing well. A specific duration (6–10 weeks) was recommended to improve global cognitive function. However, the duration (5 and 11–15 weeks) did not show an ideal effect on global cognition. The treatment time (5 weeks) was too short to exert a noticeable clinical effect on the patients with PD. During the longer duration (11–15 weeks), which was 12 weeks, patients reached a plateau where no significant clinical benefit was observed, and the results required further exploration with different treatments. Moreover, only one study each was included in the 5 and >15 weeks subgroups, and more clinical trials are needed to verify the effects of these durations. In summary, the optimal therapy for improving cognitive function was recommended to be 60–200 min per week with a duration of 6–10 weeks.

Previous studies have provided some evidence that exercise is beneficial for improving sleep quality, QOL, and daily functioning in individuals with PD (de Paula et al., 2006; Nascimento et al., 2014). Our analysis outcomes showed that mind–body excise improved sleep quality and QOL among patients with PD. Tai Chi was more sensitive to improving QOL in patients with PD. The results were persistent and stable when sensitivity analyses were conducted. Previous reviews also recommended Tai Chi, yoga, Qigong, and dance to increase the QOL of patients with PD (Sharp and Hewitt, 2014; Jin et al., 2019). In contrast, other reviews have not conclusively shown that Tai Chi or Qigong affect the QOL among PD populations (Yang et al., 2015; Yu et al., 2021) due to the limited number of studies. A meta-analysis of 25 studies indicated that regular exercise was beneficial for adults to achieve moderate to good sleep quality (Reynolds et al., 2016). Another meta-analysis of the effects of physical activity on sleep suggested that low-intensity exercise might improve sleep quality (Kredlow et al., 2015). Our meta-analysis showed that mind–body excise did not significantly improve depression symptoms or fatigue levels, consistent with the results reported by Zhang et al. (2019), who revealed that dance therapy did not significantly improve depressive symptoms or apathy levels. However, Jin et al. (2019) reported that Tai Chi, yoga, and healthy Qigong significantly reduced depressive symptoms by combining the HADS, BDI, POMS, HAMD, and SCL-90 scores. The combined BDI scores exhibited high heterogeneity. After we deleted the study by Solla et al. (2019), the heterogeneity of BDI scores was significantly reduced (*P* = 0.57, *I*^2^ = 0%), but the overall effect did not change. The PFS-16 results were persistent and stable when sensitivity analyses were conducted.

### Implications

Through our meta-analysis, we identified some potential implications for clinical practice. First, we recommend mind–body exercise as a non-pharmacological alternative treatment to improve the NMSs of patients with PD, particularly global cognitive function, sleep disturbance, and QOL. Second, among the major types of mind–body exercises, dance, Qigong, and mindfulness meditation may be more suitable for improving cognitive function, Tai Chi for improving QOL, and Qigong and mindfulness meditation for improving sleep quality. Third, a low to moderate dose (60–200 min per week) of mind–body exercise with a duration of 6–10 or >15 weeks might improve global cognitive performance, which is recommended as the optimal intensity of exercise for patients with PD presenting with or without cognitive impairment.

## Limitations

Some limitations should be emphasized. First, the number of rigorous RCTs was insufficient, and few of these studies examined NMSs as the primary outcome. Second, we tried to adopt consistent scales to evaluate NMSs, but the small number of eligible studies available in the meta-analysis may lead to limited strength and accuracy of the conclusions. Third, the effect of drugs (antidepressants, hypnotics, or anti-Parkinson’s drugs) on treatment was not analyzed because most of the included studies included patients on a stable medication regimen but did not mention the dosage or specific type of medication used. In the future, we may explore whether the intervention allows the patient to decrease the dosage of relevant medicine. Finally, very few studies reported follow-up data on the effect on non-motor manifestations, and we recommended that more trials are conducted to investigate the long-term benefit to the PD population.

## Conclusion

Based on the results of this meta-analysis, mind–body exercises are well tolerated and relatively safe. A low to moderate dose (60–200 min per week) of mind–body exercise with a duration of 6–10 or >15 weeks might improve global cognitive performance, which is recommended as the optimal intensity of exercise for patients with PD presenting with or without cognitive impairment. Due to the methodological weaknesses in the included studies and the limited sample size included in this article, our meta-analysis findings should be considered with caution. More well-designed RCTs of mind–body exercises for the treatment of NMSs of patients with PD are needed.

## Data Availability Statement

The original contributions presented in the study are included in the article/Supplementary Material, further inquiries can be directed to the corresponding authors.

## Author Contributions

KW wrote the manuscript. LL, KL, and XW contributed to the study conception. PZ and SG searched the literature. KW, PL, and LL extracted the data. XY, BJ, and PS contributed to the data acquisition. All authors have read and approved the final manuscript.

## Conflict of Interest

The authors declare that the research was conducted in the absence of any commercial or financial relationships that could be construed as a potential conflict of interest.

## Publisher’s Note

All claims expressed in this article are solely those of the authors and do not necessarily represent those of their affiliated organizations, or those of the publisher, the editors and the reviewers. Any product that may be evaluated in this article, or claim that may be made by its manufacturer, is not guaranteed or endorsed by the publisher.
