# Supplementary material for: Mind–Body Exercises for Non-motor Symptoms of Patients With Parkinson’s Disease: A Systematic Review and Meta-Analysis
Source: Front Aging Neurosci. 2021 Dec 3;13:770920. doi: 10.3389/fnagi.2021.770920 (PMC9549381; doi:10.3389/fnagi.2021.770920)
Supplement: Supplementary file 1 [file Data_Sheet_1.doc]

**Supplementary Online Content**

**Appendix 1.** Search strategy for each database

**Supplementary Figure 1.** Sensitivity analysis of included studies

**Supplementary Table 1.** Publication bias (Egg’s test)

**Appendix 1. Search strategy for each database**

**PUBMED** **search strategy**

((("parkinson disease"[MeSH Terms] OR ("parkinson*"[Title/Abstract] OR "paralysis agitan*"[Title/Abstract] OR "Parkinson's"[Title/Abstract]))) AND ((((((((((((((((((((((((mind-body[Title/Abstract]) OR (mind*[Title/Abstract])) OR (mind body*[Title/Abstract])) OR (danc*[Title/Abstract])) OR (dance therap*[Title/Abstract])) OR (tango[Title/Abstract])) OR (Tai-Ji[Title/Abstract])) OR (Tai-Chi[Title/Abstract])) OR (Tai Ji[Title/Abstract])) OR (Tai Chi[Title/Abstract])) OR (Tai ji quan[Title/Abstract])) OR (Pilates[Title/Abstract])) OR (qigong[Title/Abstract])) OR (chi kung[Title/Abstract])) OR (chi gung[Title/Abstract])) OR (qi gong[Title/Abstract])) OR (yoga[Title/Abstract])) OR (yogic[Title/Abstract])) OR (asana[Title/Abstract])) OR (pranayama[Title/Abstract])) OR (dhyana[Title/Abstract])) OR (dharana[Title/Abstract])) OR (Baduanjin[Title/Abstract])) OR (wuqinxi[Title/Abstract]))) AND ((((randomized controlled trial[Title/Abstract]) OR (controlled clinical trial[Title/Abstract])) OR (random*[Title/Abstract])) OR (trial[Title/Abstract]))

**EMBASE search strategy**

#1. 'parkinson disease'/exp

#2. 'parkinson*':ab,ti

#3. 'paralysis agitan*':ab,ti

#4. #1 OR #2 OR #3

#5. 'mind-body':ab,ti

#6. 'mind*':ab,ti

#7. 'mind body*':ab,ti

#8. 'danc*':ab,ti

#9. 'dance therap*':ab,ti

#10. 'tango':ab,ti

#11. 'tai-ji':ab,ti

#12. 'tai-chi':ab,ti

#13. 'tai ji quan':ab,ti

#14. 'pilates':ab,ti

#15. 'qigong':ab,ti

#16. 'chi kung':ab,ti

#17. 'chi gung':ab,ti

#18. 'qi gong':ab,ti

#19. 'yoga':ab,ti

#20. 'yogic':ab,ti

#21. 'asana':ab,ti

#22. 'pranayama':ab,ti

#23. 'dhyana':ab,ti

#24. 'dharana':ab,ti

#25. 'baduanjin':ab,ti

#26. 'wuqinxi':ab,ti

#27. #5 OR #6 OR #7 OR #8 OR #9 OR #10 OR #11 OR #12 OR #13 OR #14 OR #15 OR #16 OR #17 OR #18 OR #19 OR #20 OR #21 OR #22 OR #23 OR #24 OR #25 OR #26

#28. 'randomized controlled trial':pt

#29. 'controlled clinical trial':pt

#30. 'random*':ab

#31. 'trial':ab

#32. #28 OR #29 OR #30 OR #31

#33. #4 AND #27 AND #32

**COCHRAIN search strategy**

ID Search

#1 MeSH descriptor: [Parkinson Disease] explode all trees

#2 (parkinson*):ti,ab,kw OR (paralysis agitan*):ti,ab,kw OR (Parkinson's):ti,ab,kw (Word variations have been searched)

#3 #1 or #2

#4 (mind body):ti,ab,kw OR (mind*):ti,ab,kw OR (mind body*):ti,ab,kw OR (danc*):ti,ab,kw OR (dance therap*):ti,ab,kw (Word variations have been searched)

#5 (tango):ti,ab,kw OR (Tai-Ji):ti,ab,kw OR (Tai-Chi):ti,ab,kw OR (tai ji quan):ti,ab,kw OR (Pilates):ti,ab,kw (Word variations have been searched)

#6 (qigong):ti,ab,kw OR (chi kung):ti,ab,kw OR (chi gung):ti,ab,kw OR (qi gong):ti,ab,kw OR (yoga):ti,ab,kw (Word variations have been searched)

#7 (yogic):ti,ab,kw OR (asana):ti,ab,kw OR (pranayama):ti,ab,kw OR (dhyana):ti,ab,kw OR (dharana):ti,ab,kw (Word variations have been searched)

#8 (Baduanjin):ti,ab,kw OR (wuqinxi):ti,ab,kw (Word variations have been searched)

#9 #4 or #5 or #6 or #7 or #8

#10 #3 and #9

**Supplementary Figure 1. Sensitivity analysis of included studies**

**MoCA outcome**


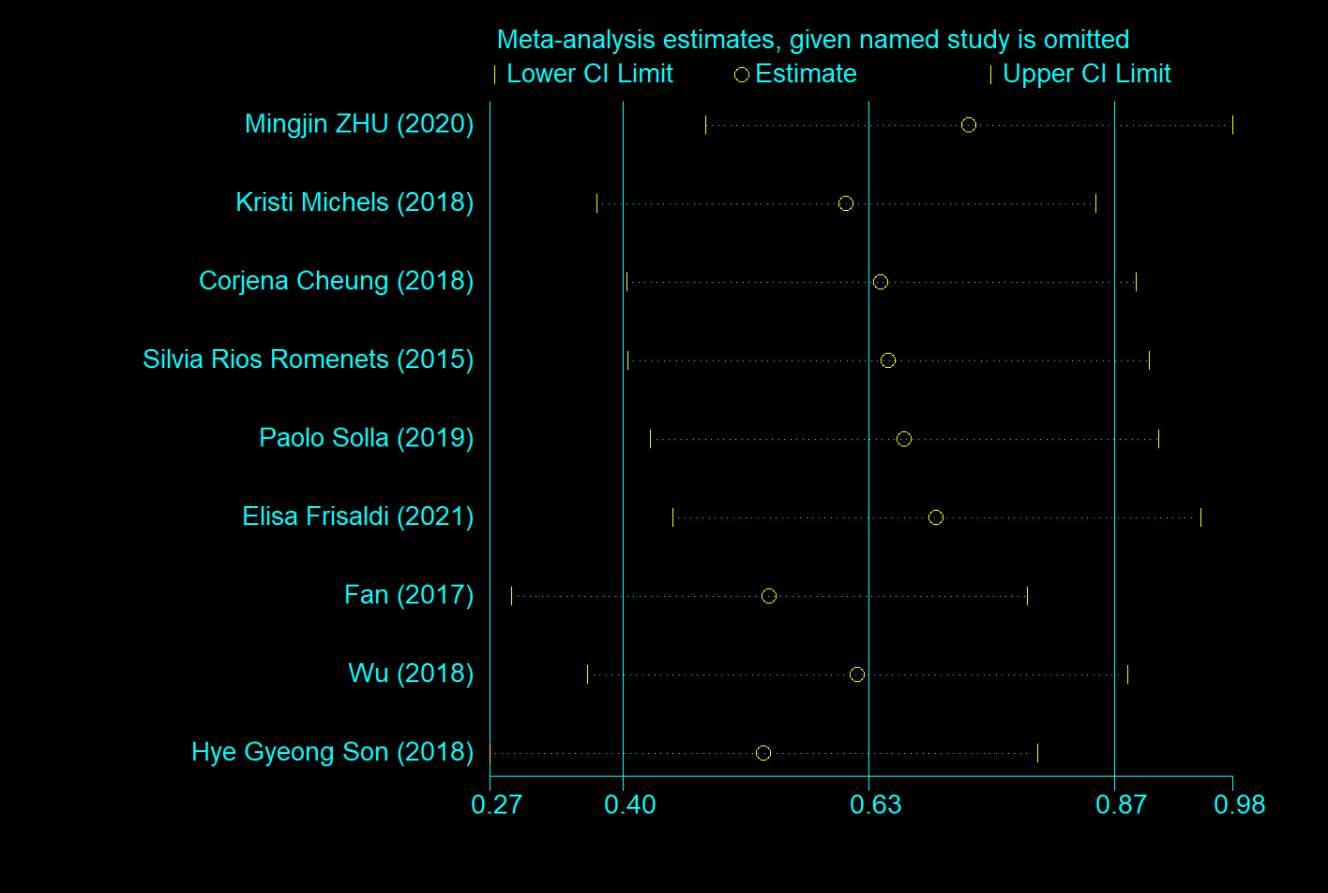


**Supplementary Table 1. Tests for Publication Bias (Egger’s test)**

**MoCA outcome**

**Egger's test**

| Std_Eff | Coef. | Std. Err. | t | P>|t| | [95% Conf. Interval] | |
| --- | --- | --- | --- | --- | --- | --- |
| slope | -.6110527 | 1.824225 | -0.33 | 0.747 | -4.924659 | 3.702553 |
| bias | 2.651293 | 2.239794 | 1.18 | 0.275 | -2.644977 | 7.947564 |
